# Promising medium-term results of anterior approach with an anatomical short stem in primary hip arthroplasty

**DOI:** 10.1186/s10195-021-00567-x

**Published:** 2021-03-06

**Authors:** Fabrizio Rivera, Alessandro Bardelli, Andrea Giolitti

**Affiliations:** 1 Orthopedics and Trauma Department, SS Annunziata Hospital, ASL CN1, Savigliano (CN), Italy; 2 Orthopeadics and Traumatology Department, Faculty of Medicine and Surgery, CTO Hospital, University of Turin, Turin, Italy

**Keywords:** Anatomic hip stem, Short hip stem, Direct anterior approach, Standard table, Leg positioner

## Abstract

**Background:**

In the last decade, the increase in the use of the direct anterior approach to the hip has contributed to the diffusion of the use of short stems in orthopedic surgery. The aim of the study is to verify the medium-term clinical and radiographic results of a cementless anatomic short stem in the anterior approach to the hip. We also want to verify whether the use of the standard operating room table or the leg positioner can affect the incidence of pre- and postoperative complications.

**Materials and methods:**

All total hip arthroplasty patients with a 1-year minimum follow-up who were operated using the MiniMAX stem between January 2010 and December 2019 were included in this study. Clinical evaluation included the Harris Hip Score (HHS), Western Ontario and McMaster Universities Hip Outcome Assessment (WOMAC) Score, and Short Form-36 (SF-36) questionnaires. Bone resorption and remodeling, radiolucency, osteolysis, and cortical hypertrophy were analyzed in the postoperative radiograph and were related to the final follow-up radiographic results. Complications due to the use of the standard operating room table or the leg positioner were evaluated.

**Results:**

A total of 227 patients (238 hips) were included in the study. Average age at time of surgery was 62 years (range 38–77 years). Mean follow-up time was 67.7 months (range 12–120 months). Kaplan–Meier survivorship analysis after 10 years revealed 98.2% survival rate with revision for loosening as endpoint. The mean preoperative and postoperative HHS were 38.35 and 94.2, respectively. The mean preoperative and postoperative WOMAC Scores were 82.4 and 16.8, respectively. SF-36 physical and mental scores averaged 36.8 and 42.4, respectively, before surgery and 72.4 and 76.2, respectively, at final follow-up. The radiographic change around the stem showed bone hypertrophy in 55 cases (23%) at zone 3. In total, 183 surgeries were performed via the direct anterior approach (DAA) on a standard operating room table, and 44 surgeries were performed on the AMIS mobile leg positioner. Comparison between the two patient groups did not reveal significant differences.

**Conclusion:**

In conclusion, a short, anatomic, cementless femoral stem provided stable metaphyseal fixation in younger patients. Our clinical and radiographic results support the use of this short stem in the direct anterior approach.

**Level of evidence:**

IV.

## Introduction

Short stems have been introduced into surgical practice to achieve greater preservation of proximal bone stock and stability than those of conventional stems [1–4]. In the last decade, the increase in the use of the direct anterior approach to the hip has contributed to the diffusion of short stems in orthopedic surgery. In fact, short stems are easier to insert when using an anterior approach [5, 6]. In this case, their use can reduce the risk of intraoperative fracture of the femur and facilitate surgery [7, 8]. Ideally, compared with the standard stem, the shortened stem should have greater metaphyseal filling providing adequate metaphyseal fixation and proper alignment. Theoretically, a short anatomic stem should meet this requirement better than a short straight stem [9, 10]. It is unclear whether the short stem has a higher survival rate than the standard stem. Furthermore, it is not clear how reliable a short anatomic stem is and what indications and results it can provide. The aim of the study is to verify the medium-term clinical and radiographic results of a cementless anatomic short stem in the anterior approach to the hip. We also want to verify whether the use of the standard operating room table or the leg positioner can affect the incidence of pre- and postoperative complications. We hypothesize that the anatomic short stem provides excellent medium-term stability and survival results regardless of the type of operating table used as a support to the surgical technique.

## Patients and methods

All total hip arthroplasty patients with 1-year minimum follow-up who were operated using the MiniMAX stem (Medacta International, Castel San Pietro, Switzerland) implant at SS Annunziata Hospital, Savigliano (CN), Italy, between January 2010 and December 2019 were included in this study.

The exclusion criteria for the use of the MiniMAX stem were age > 80 years, fractures, osteolytic lesions, body mass index (BMI) > 35 kg/m^2^. These exclusion criteria have always been maintained at our department for the use of short stems.

Clinical data (gender, age, weight, and height) and comorbidities (cardiovascular, respiratory, gastrointestinal, nutritional, endocrine, genitourinary) were collected retrospectively from medical records and outpatient control cards.

Radiographic data were taken from the hospital database (picture archiving and communication system). Decision whether to use the MiniMAX stem was made by the orthopedic surgeon, in compliance with the exclusion criteria, operating on the basis of age, fragility, bone morphology, and level of activity of the patient.

MiniMAX stem is anatomically designed, cementless, collarless, and made of titanium-niobium alloy (Ti-6Al-7Nb). It is round-coated with hydroxyapatite, Ra 80 μm, all along the shaft and Ti coating, Ra 300 μm, in the proximal two-thirds of the shaft. The neck has a 127° neck-shaft angle with an anteversion of 9°. MiniMAX stems can be classified as type 6 according to Khanuja et al. [11] because they are curved, anatomic stems that match the proximal femoral endosteal geometry. A cementless acetabular cup (Versafit, Medacta International, Castel San Pietro, Switzerland) with ceramic–ceramic bearing was used in all cases. The femoral head diameter depended on cup size, 32 mm for cup diameter 48 mm or less, and 36 mm for cup diameter 50 mm or more. All surgeries were done using direct anterior approach [12, 13]. The same surgical times were recorded in all patients with the support of the standard operating room table or the AMIS mobile leg positioner (Medacta International, Castel San Pietro, Switzerland).

Primary outcomes were stem revision for aseptic loosening and all-cause stem revision. Second outcomes were clinical and radiographic results at medium-term follow-up.

Patient follow-up was performed, at 6 weeks, 3 months, and then annually postsurgery.

Clinical evaluation included the Harris Hip Score (HHS) [14], Western Ontario and McMaster Universities Osteoarthritis Index (WOMAC) Score [15], and SF-36 questionnaires [16].

During preoperative and postoperative radiographic controls, anteroposterior and axial hip radiographs were taken with the foot in a neutral rotational position. Femoral geometries were categorized according to the Door classification system [17] using preoperative anteroposterior radiographs of the hip. The calcar-to-canal ratio was calculated by dividing the canal width, measured at 10 cm below the lesser trochanter, by the calcar width, measured at the middle level of the lesser trochanter, as previously described [17]. Femurs with a ratio of 0–0.5 were considered type A, 0.5–0.75 as type B, and 0.75–1 as type C [18, 19].

**Fig. 1:**
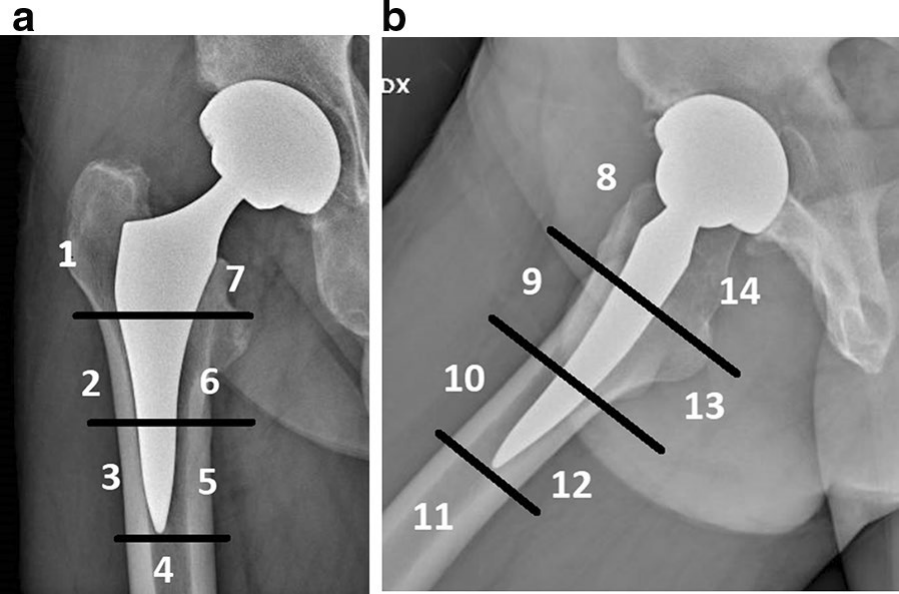
Radiographic follow-up and definition of Gruen’s periprosthetic zones in anteroposterior view (**a**) and axial view (**b**)

Alignment of the stem was considered neutral when the vertical axis of the stem was between 0° and 2° with respect to the femoral shaft axis. A varus–valgus alignment was classified as mild in case of misalignment between 2° and 5° and severe when the misalignment of the stem was > 5°. The most recent radiographs were compared with the first postoperative clinic radiographs to evaluate bony remodeling and changes in implant positioning. Stem subsidence was diagnosed in the presence of a stem sinking > 3 mm, measured on a perpendicular line drawn from the greater trochanter to the lateral edge of the implant. Implant loosening was diagnosed in the presence of subsidence and/or axial deviation in varus/valgus. We judged early subsidence or axis deviation within 6 months of surgery, late when observed after 6 months postoperation. According to the modified zones described by Gruen [20] (Fig. 1), bone resorption and remodeling, radiolucency, osteolysis, and cortical hypertrophy were analyzed in the postoperative radiographs and were related to the final follow-up radiographic results.

Finally, to evaluate the possible impact on the results of the only variable present in the study group, we divided the population observed into patients undergoing surgery with the support of the standard operating room table (group A) or the AMIS mobile leg positioner (group B). The two groups were compared for demographic data and for final clinical and radiographic results.

Patient demographics and clinical and radiographic outcomes are presented as descriptive statistics. Mean and standard deviations are provided when applicable. Statistics were performed using MedCalc Software Version 11.1 (MedCalc Software, Mariakerke, Belgium)*.*

The paired *t* test was used to compare preoperative and postoperative values at the final follow-up of HHS, WOMAC Scores, and SF-36 physical and mental scores. Wilcoxon signed-rank test was used to compare characteristics of patients undergoing surgery with the support of the standard operating room table (group A) with those undergoing surgery with the support of the AMIS mobile leg positioner (group B). Statistical significance was set at *p* value < 0.05. Kaplan–Meier survival analysis was performed with the end point of stem revision for loosening.

## Results

**Fig. 2:**
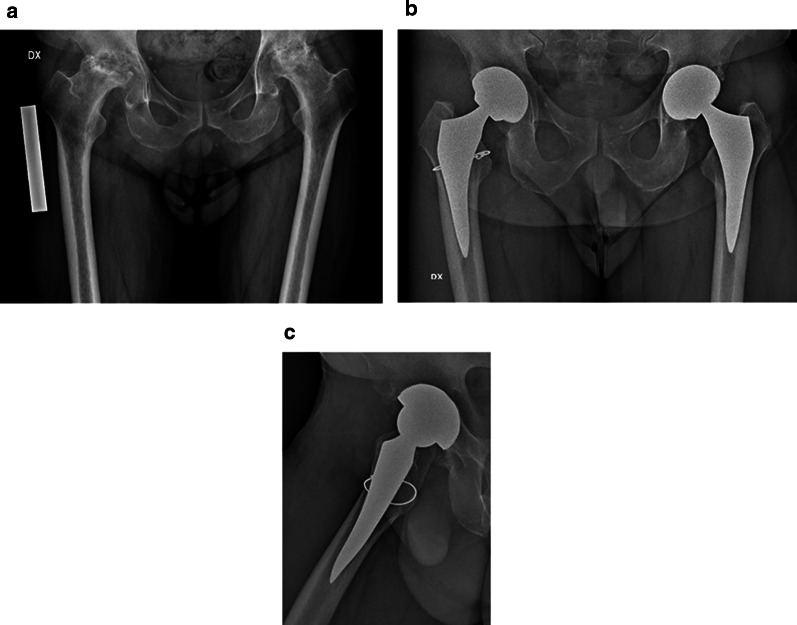
Bilateral avascular necrosis of femoral head in a 46-year-old patient (**a**). Five years radiographic follow-up: anteroposterior view (**b**) and axial view of right (**c**) and left (**d**) hips. Undisplaced fracture of calcar region treated with preventive cerclage on right hip

We retrospectively reviewed a group of 237 consecutive patients who underwent total hip arthroplasty from January 2009 to October 2019, of whom 11 were operated bilaterally in a single procedure (Fig. 2), for a total of 248 hips; nine patients lost to 3-month follow-up and one patient who died from causes not related to surgery were excluded from the study. Of the remaining 227 patients (238 hips), 145 (63.8%) were female and 82 (36.2%) were male. The average age at the time of surgery was 62 years (range 38–77 years). The preoperative diagnosis was primary osteoarthritis in 175 cases (9 bilateral), avascular necrosis of the femoral head in 28 cases (1 bilateral), rheumatoid arthritis in 9 cases (1 bilateral), traumatic osteoarthritis in 12 cases, and other causes in 3 cases. Demographic data of patients are summarized in Table 1. Mean follow-up time was 67.7 months (range 12–120 months).

**Table 1:** Demographic data of patients

Parameters	Values
No. of patients	227
No. of hips	238
Gender (male/female)	82 (36.2%)/145 (63.8%)
Mean age (years)	62 (38–77)
Mean weight (kg)	64 (57–82)
Mean height (cm)	159 (145–192)
Mean BMI (kg/m^2^)	24.1 (15.5–33.3)
Comorbidities (*n*)	1.3 (1–4)
Mean follow-up (months)	67.7 (12–108)
Diagnosis (*n*patients)	
Osteoarthritis	175
Avascular necrosis	28
Rheumatoid arthritis	9
Traumatic osteoarthritis	12
Other causes	3
Surgical technique (*n*.patients)	
Standard operating room table	183
AMIS mobile leg positioner	44

**Fig. 3:**
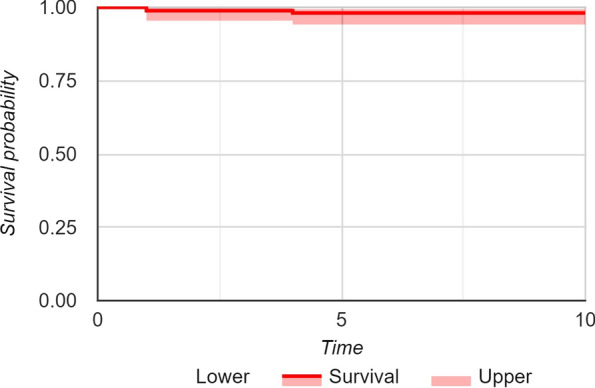
Kaplan–Meier survivorship analysis after 10 years. Survival rate of 98.2% (95% CI, 94.1–96.1%) with revision for loosening as endpoint

Three stems were revised. One cortical perforation was observed postoperation on postoperative radiographic control and then revised, one clinically symptomatic early subsidence was revised 5 months after surgery, and one aseptic loosening was revised 4 years after surgery. We also observed two periprosthetic fractures B1 according to the Vancouver classification. The intraoperative complications observed, in addition to the case of cortical perforation, were five undisplaced fractures of the calcar region treated with preventive cerclage (Fig. 2). One early infection was treated with surgical washing and head and liner revision followed by antibiotic therapy. No dislocations were observed. (Table 2) Kaplan–Meier survivorship analysis after 10 years revealed 98.2% (95% CI, 94.1–96.1%) survival rate with revision for loosening as endpoint (Fig. 3).

**Table 2:** Major and minor complications

Complications	
Major complications (stem revision) Cortical perforations Subsidence Aseptic loosening Periprosthetic fractures (ORIF) Minor complications Calcar fractures (intraoperative cerclage) Subsidence Conservative treatment ≤ 3 mm Conservative treatment > 3 mm Early infection	3 (1.2%) 1 1 1 2 18 (7.5%) 5 (2.1%) 3 11 (4.6%) 3 1

Comparison of mean preoperative and postoperative HHS, WOMAC Score, and the SF-36 questionnaires at the final follow-up revealed significant differences. The mean preoperative and postoperative HHS were 38.35 (11–64, SD 16.3) and 94.2 (68–100, SD 9.1), respectively. The mean preoperative and postoperative WOMAC Scores were 82.4 (58–96, SD 13.1) and 16.8 (0–49, SD 13.9), respectively. SF 36 physical and mental scores averaged 36.8 (range 32–44, SD 7.3) and 42.4 (range 34–48, SD 8.4), respectively, before surgery, and 72.4 (68–96, SD 8.1) and 76.2 (66–94, SD 10.3), respectively, at final follow-up (Table 3).

**Table 3:** Comparison of the clinical and radiographic results between preoperative and postoperative values

Parameters	Preop. values (range, SD)	Values at final follow-up	*p*
Harris Hip Score (HHS) WOMAC Score	38.35 (11–64, 16.3) 82.4 (58–96, 13.1)	94.2 (68–100, 9.1) 16.8 (0–49, 13.9)	0.005 0.015
SF-36 questionnaires			
Physical Mental	36.8 (range 32–44, 7.3) 42.4 (range 34–48, 8.4)	72.4 (68–96, 8.1) 76.2 (66–94, 10.3)	0.031 0.029

**Fig. 4:**
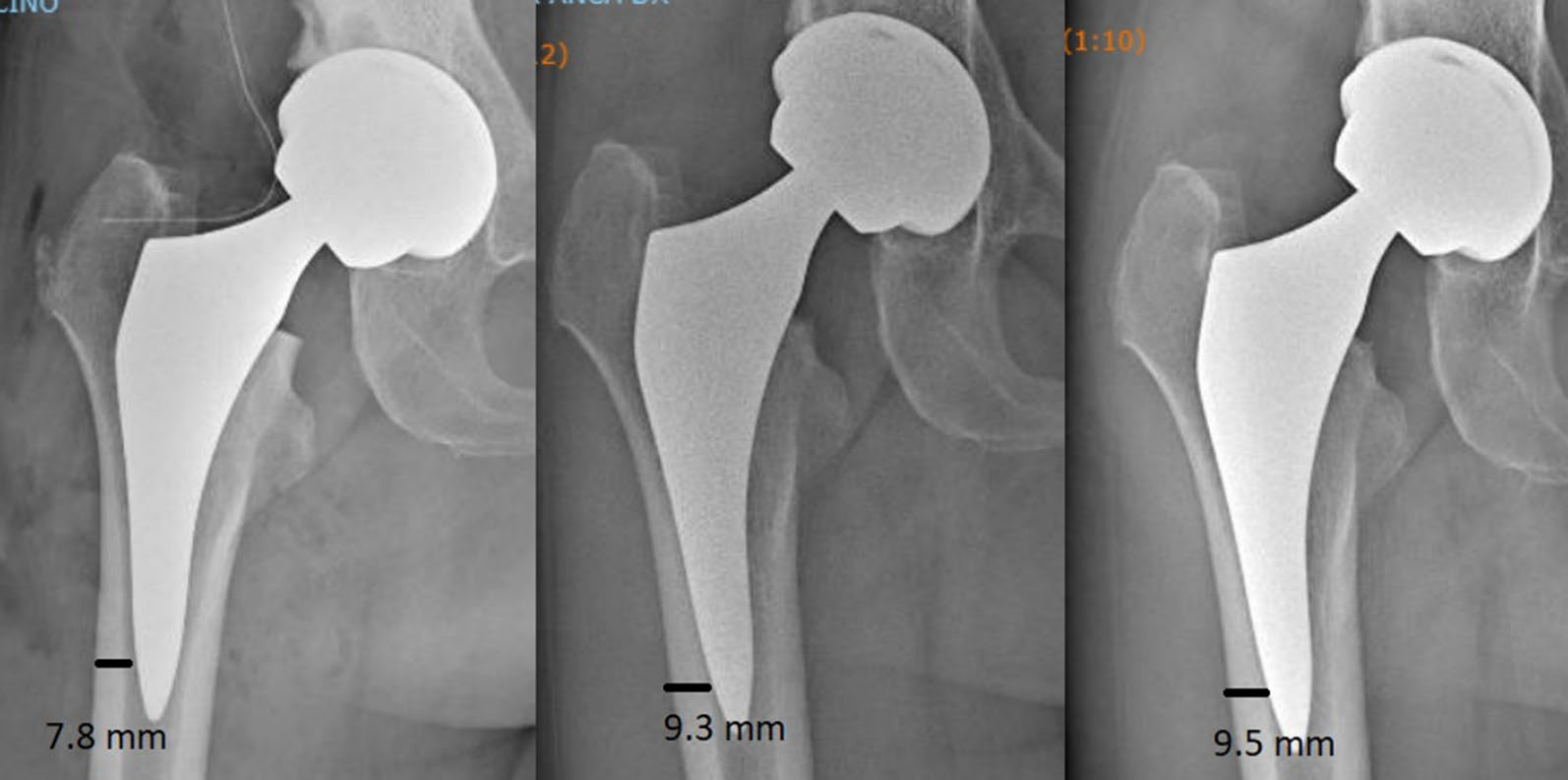
Radiographic change around stem in a 62-year-old patient: postoperative radiograph (left), 1 year follow-up radiograph showing bone cortical hypertrophy in zone 3 (central), 5 years follow-up radiograph with evidence of stabilization of cortical hypertrophy

According to Dorr classification [17], 105 hips (44%) were graded as Dorr A, 125 hips (52%) as Dorr B, and 8 hips (4%) as Dorr C. In 189 (79%) cases, the alignment of the stem was considered neutral, in 41 (17%) cases it was considered mild varus–valgus, and in 8 (3%) cases severe varus–valgus. We did not observe any correlation between the morphology of the femoral canal and the varus–valgus positioning of the stem. In total, the varus–valgus positioning (medium or severe) was observed in 23 (22%) hips type Dorr A and in 26 (21%) hips type Dorr B. The radiographic change around the stem showed bone trabeculae hypertrophy in 55 cases (23%) at zone 3 (Fig. 4), in 17 cases (7%) at zone 5, and 4 cases (1.6%) at zone 4. There was grade 1 stress shielding (calcar round-off) in four cases (1.6%). No hypertrophy at zones 8–14 (lateral view) were observed. No correlations between varus–valgus stem positioning and distal cortical hypertrophy were observed.

Stem subsidence > 3 mm, in addition to the one symptomatic case revised, was observed three more times (4 mm, 7 mm, and 10 mm, respectively). In all three cases, absence of pain and tolerated leg length discrepancy did not compromise the good final functional result (Table 4).

**Table 4:** Radiographic results at last follow-up

Femoral geometries	Dorr A 105 (44%)	Dorr B 125 (52%)	Dorr C 8 (4%)
Stem alignment *n* (%)	Neutral 189 (79%)	Varus–valgus < 5° Varus 36 (15%) Valgus 5 (2%)	Varus–valgus > 5° Varus 8 (3%) Valgus 0
Stem subsidence *n* (%)	≤ 3 mm 11	> 3 mm revised 1	> 3 mm not revised 3
Proximal stress shielding *n* (%) Distal cortical hypertrophy	Zone 1 4 (1.6%) Zone 3 55 (23%)	Zone 4 17 (7%)	Zone 5 4 (1.6%)

Finally, 183 (80.6%) surgeries (group A, 11 bilateral) were performed via the DAA using a standard operating room table and 44 (19,4%) surgeries (group B) were performed using the AMIS mobile leg. Comparison between the two patient groups did not reveal significant differences between demographic characteristics and the incidence of complications (Table 5).

**Table 5:** Demographic and clinical characteristics of patients undergoing surgery with support of standard operating room table (group A) or AMIS mobile leg positioner (group B)

Parameters	Group A	Group B	*p*
No. of patients	183	44	
No. of hips	194	44	
Gender (male/female)	110/73	22/12	0.54
Mean age (years)	63 (38–77)	62 (41–80)	0.48
Mean BMI (kg/m^2^)	24.2 (15.5–33.3)	23.8 (14.9–33.1)	0.46
Mean follow-up	67.9 (12–98)	65.9 (12–108)	0.39
No. of revisions	2	1	
No. of intraoperative cerclage	4	1	

## Discussion

This study was undertaken to evaluate a medium-term outcome of a cementless short anatomic stem in terms of survival and low complication rates. For this reason, we retrospectively evaluated clinical and radiographic results and a mean follow-up of 67.7 months (range 12–108 months) in 227 patients (238 hips) treated by anterior approach.

The use of an anatomic short stem is not widespread. If the traditional anatomic stem is well defined as a posteriorly bowed portion of the canal and an anteverted neck portion [11], the definition of an anatomic stem has become less precise in the case of a short stem. The Joint Implant Surgery and Research Foundation (JISRF) defines bulky stems or fit and fill (type 3B) anatomic stems (left and right) with an anteversion rate (6–12°) incorporated into a one-piece neck/stem configuration [21].

Kim et al. [22] reported long-term results of 638 hips treated with a titanium stem with a metaphyseal shaped anatomical portion, lateral flare, and a circumferential porous coating. Femoral stem features included a neck-shaft angle of 126°, a femoral neck anteversion of 8°, and a femoral neck diameter of 12 mm. At the mean follow-up of 15.8 years, they reported a mean Harris Hip Score of 94 points and a mean WOMAC Score of 15 points. Survival rate was 100% with aseptic loosening as endpoint. Similar results using the same stem have been reported by Cinotti et al. [23] after a revision of 72 patients at 9 years follow-up. They reported a mean Harris Hip Score of 88, a mean WOMAC Score of 24 points, and a survival rate of 100%.

Koyano et al. [24] reported their experience with an anatomic stem with a shortened and tapered distal portion. The stem has a grit-blasted surface with hydroxyapatite coating on the proximal third. In a group of 36 patients, the authors reported an improvement in the Japanese Orthopaedic Association (JOA) Hip Score from 43 to 14 points at 9.2 years follow-up.

Patil et al. [26] studied a modified 3° biplanar taper geometry stem. The stem was 80 mm in length, anatomic, with 5° of anteversion. It was composed of titanium alloy, and the 80% proximal portion of the stem was circumferentially coated with porous plasma spray. A mean 42.8-month radiographic follow-up assessment was performed for 77 patients. There were no radiologic signs of migration, subsidence, or loosening in this series. No clinical results were reported.

Overall, few papers describe the results of uncemented short stems [22–26]. To this problem in evaluating the results we must add the difficulty of identifying the characteristics of the anatomic stems, often classified as such but lacking the fundamental characteristics.

McTighe et al. [11] included in type 3b of the Joint Implant Surgery and Research Foundation (JISRF) classification, a left/right implant, with lateral flare standard neck with 12 degrees of anteversion. The lack of the posterior bowed portion of the canal is in our opinion a feature that prevents the definition of an anatomic stem, a definition that cannot be justified by the mere presence of an anteversion of the neck. In the lateral plane, an anatomic stem bows posteriorly in the metaphysis and anteriorly in the diaphysis. For this reason, the MiniMAX stem is one of the few short stems that can be included in this definition.

The anatomical stem design bends posteriorly so that it can be adapted to the proximal femoral endosseous geometry and achieve maximum contact following the natural shape of the femoral canal [11, 22, 25–27]. This design fills the metaphysis to the maximum. In the case of a standard anatomic stem, the canal is also filled for the first part of the shaft. The distal part of the stem in fact also fills the diaphysis, allowing an increase in primary stability. For this reason, the anatomic stem should provide excellent initial stability and reliable bone growth [25–28]. The three main concerns are (1) the high variability of the femoral canal anatomy in the population, so the stem may not fit one of the other patients; (2) a possible high incidence of periprosthetic fractures due to the posterior arch; and (3) a high incidence of thigh pain due to closed fit [3, 8, 22]. The short anatomic stem, lacking the portion that fills the first part of the shaft, could theoretically have less primary stability but should result in a lower incidence of thigh pain [8–10].

The high variability of the femoral canal anatomy in the population is demonstrated in our opinion by our postoperative radiographic results. The 22% varus or valgus placement, even if moderate, indicates the adaptability of this stem design to the femoral canal and not vice versa.

The incidence of periprosthetic fractures in our study group was very low. Further studies are needed to determine whether, as with the standard anatomic stem, the short anatomic stem presents a higher risk of periprosthetic fracture.

Regarding the possibility of an increased risk of thigh pain, the clinical and radiographic findings allow for some considerations. The particular anatomical design and the lateral flare of the metaphyseal portion certainly provides a complete fit of the proximal portion of the stem. In general, short stems have a significantly smaller bone contact area than standard stems and, as a result, may have less torsional and axial resistance. A solution proposed in the literature to overcome the problem is to spare the femoral neck, by means of a high osteotomy [3, 4, 29–31]. The short neck-sparing stems provide additional axial and torsional stability and reduce stress at the bone interface of the implant. In the case of the short anatomic stem, it is the metaphyseal geometry that maintains stability [25, 32]. In our experience, the adaptation and maximum filling of the metaphyseal canal, optimizing primary stability, did not necessitate a neck-sparing surgical technique.

Despite the incidence in our study group of cortical hypertrophy in Gruen zones 3 and 5 up to 23%, from a clinical point of view we have not found any correlation between hypertrophy of the distal cortex and thigh pain. Among the causes of cortical bone hypertrophy discussed in the literature, the first is the transfer of the distal load by the stem. The abnormal load transmitted from the stem to the bone, in particular overload around the tip of the stem rather than the calcar area, can cause bone resorption in the calcar region and the development of a hypertrophy reaction in the distal region of the stem [3, 4, 33].

Another cause considered is the design and coating of the stem, in particular in the distal portion where excessive bone ingrowth and excessive apex–cortical bone contact can generate hypertrophy [30, 34]. The absence of areas bone density reduction in the proximal region, the anatomical design of the apex of the stem, and the titanium and hydroxyapatite coating only in the proximal two-thirds of the surface make us exclude these two causes. A third cause of cortical hypertrophy described in the literature is stiffness of short stems [32, 34]. This may be due to a widened axial section of the stem and therefore an enlarged implant rigidity of the shorter stem compared with standard stem sections [32, 35]. We believe that this possibility is the most plausible to explain the presence of distal bone hypertrophy in our study group. On the other hand, the lateral flare and the anatomical design of the proximal portion allow an excellent fill of the metaphyseal region and prevent a distal load transfer. Distal hypertrophy observed in our study group for this reason is quantitatively limited and does not cause thigh pain. The evidence of the phenomenon in the first year of follow-up and the subsequent stabilization of the media observed in our cases further show how, in the case of the MiniMAX stem, the hypertrophy of the cortex in zone 3 and 5 is a bone adaptation to the new stiffness conditions of the proximal femoral portion, which quickly finds a balance between the forces at play (Fig. 4).

Direct anterior approach was used in all cases of our group of study. The direct anterior approach was originally proposed on a specialized traction/fracture table (traction table) [36]. Over time, the high incidence of intraoperative fractures has also been attributed to the use of traction of the affected limb [37]. For this reason, the support of the standard operating room table has been proposed in the literature [38–40]. Currently, no support has shown better results than the other. Ultimately it is the organization of the operating room and the surgeon’s experience that determine the choice of the type of operating table used [40, 41]. Also in our experience, we have not found any differences in the outcome and incidence of intraoperative complications using the two types of operative support (Table 5). The use of the standard operating table, with the patient in the supine position and free limbs, has the advantage of directly evaluating the length of the lower limbs by comparing the operated limb with the contralateral. The leg positioner has the advantage of easier preparation of the sterile draping and easier access to the hip of the intraoperative fluoroscopy. It also requires the training of an operating room nurse to use the positioner but requires one fewer assistant surgeon, who is not required for limb and hip mobilization, unlike with the standard table.

There are several limitations in this study. First, although the data were collected prospectively, the study was retrospective in design, not randomized. Second, we did not use radiostereometric analysis to evaluate for stem subsidence; radiostereometric analysis is known to be more precise than manual techniques of measurement [42]. Similarly, we did not use dual-energy X-ray absorptiometry to measure bone mineral density, but we used manual measurement to evaluate cortical hypertrophy [43]. Third, our study group is characterized by relatively young patients. This is due to the choice of using a short stem in selected patients. The anterior approach is performed by us by intraoperative fluoroscopic control in patients younger than 75 years. The selection criteria may partially influence our results. The literature reports a higher rate of intraoperative complications in the case of an anterior approach [44–46]. However, the selection of the younger patient, with a better bone stock than the elderly patient, should report a risk of intraoperative complications equal or similar to that of the more common posterolateral approach.

In conclusion, a short, anatomic, cementless femoral stem provided stable metaphyseal fixation in younger patients. Our clinical and radiographic results support the use of this short stem in the direct anterior approach. Distal cortical hypertrophy sometimes detectable in the distal region at early follow-up probably represents adaptation of the femur to the new rigidity conditions, but the hypertrophy is not symptomatic and does not correspond to proximal stress-shielding zones. Further research is needed to assess the long-term results of the MiniMAX stem in comparison with other short cementless stems in total hip arthroplasty.

## Data Availability

Paper copy of the database is available at SS Annunziata Hospital, Savigliano (CN), Italy.
